# Early Determination of Tacrolimus Concentration–Dose Ratio Identifies Risk of Allograft Loss in Kidney Transplantation

**DOI:** 10.1016/j.ekir.2025.02.014

**Published:** 2025-02-25

**Authors:** Christophe Masset, Marine Lorent, Clarisse Kerleau, Claire Garandeau, Aurélie Houzet, Simon Ville, Diego Cantarovich, Gilles Blancho, Magali Giral, Jacques Dantal, Nantes DIVAT Consortium, Gilles Blancho, Julien Branchereau, Diego Cantarovich, Agnès Chapelet, Jacques Dantal, Florent Delbos, Clément Deltombe, Lucile Figueres, Charles Ronsin, Thibault Letellier, Clémence Petit, Claire Garandeau, Magali Giral, Caroline Gourraud-Vercel, Laurent Nicolet, Christine Kandel-Aznar, Ismaël Chelghaf, Clarisse Kerleau, Christophe Masset, Aurélie Meurette, Karine Renaudin, Simon Ville, Alexandre Walencik

**Affiliations:** 1 Service de Néphrologie et Immunologie Clinique, Institut de Transplantation Urologie Néphrologie (ITUN), CHU Nantes, Nantes, France; 2 Center for Research in Transplantation and Translational Immunology, UMR 1064, Nantes Université, INSERM, Institute of Transplantation Urology and Nephrology, CHU Nantes, Nantes, France

**Keywords:** allograft survival, kidney transplantation, tacrolimus toxicity

## Abstract

**Introduction:**

Fast tacrolimus–metabolizing kidney transplant recipients (KTRs) (i.e., tacrolimus trough-level/total daily dose [C0/D < 1.05]) have poorer allograft function; however, their identification in a real-life setting is challenging. We investigated the reproducibility of tacrolimus metabolic status during the first months after transplantation and its association with long-term allograft outcomes.

**Methods:**

All KTRs between 2000 and 2019 with a functional allograft at 1 month and receiving tacrolimus in our center were included. Fast or slow tacrolimus metabolizers were classified according to the time spent with a C0/D < 1.05 (> 75% = High, < 25% = Low) at various time points posttransplantation. We first determined the earliest accurate time for patient categorization by investigating C0/D variability during the first months. Second, a multivariate cause-specific Cox model studying allograft outcomes was performed in groups identified by their status determined from the earliest accurate timepoint after transplantation.

**Results:**

Among 1979 patients included in the analysis, 2 months was the earliest accurate timepoint to determine High patients (85% of High patients identified at 2 months remained High long-term, Brier score = 0.06). Multivariate analysis revealed that High patients determined at 2 months (*n* = 499) had a significantly higher risk of allograft loss (cause-specific hazard ratio [CS-HR] = 2.00, 95% confidence interval [CI] = 1.48–2.69) and allograft rejection (CS-HR = 1.71, 95% CI = 1.15–2.54) than Low patients after adjustment for confounding factors. Moreover, allograft function was lower in High patients (46.7 vs. 52.9 ml/min, at 3 years, *P* < 0.0001) with a higher proportion of chronic vascular lesions at 1 year.

**Conclusion:**

C0/D is a simple and pragmatic tool capable of identifying patients at risk of rejection and allograft failure as early as the second month posttransplantation.

## Graphical abstract

**Figure:**
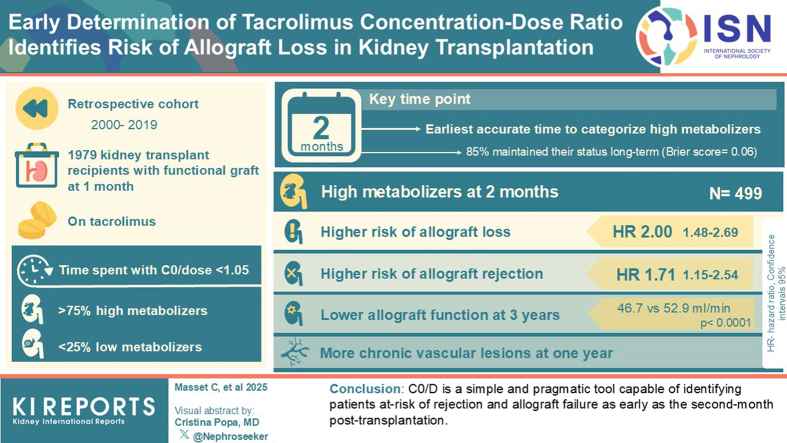

See Commentary on Page 1326

Calcineurin inhibitors (CNIs) are currently a cornerstone of maintenance therapy in kidney transplant recipients (KTR).^1^^,^^2^ The monitoring and adjustment of tacrolimus dosing is important because its therapeutic window is narrow with high intraindividual and interindividual variability.^3^ Indeed, low tacrolimus concentrations are associated with an increased risk of allograft rejection,^4^ whereas high CNI exposure may result in nephrotoxicity.^5^

Tacrolimus is metabolized mainly via cytochrome CYP3A5 and varies considerably among individuals because of genetic polymorphisms. Nevertheless, genetic screening of KTRs remains questionable, because adapting tacrolimus to CYP3A5 expression is not associated with improved allograft outcomes.^6^^,^^7^ In addition, CNI metabolism depends on multiple extrinsic factors (drug interactions and patient distribution volume).^8^^,^^9^

Recently, the ratio between the tacrolimus trough level, C0 (in ng/ml) on tacrolimus total daily dose, D (in mg), defined as C0/D, has been described as an efficient and simple tool to assess tacrolimus metabolism.^10^ C0/D is not a direct reflection of tacrolimus metabolism but a combination of drug absorption and metabolism. According to their C0/D ratio, KTRs can be categorized as “high tacrolimus metabolizers” (C0/D < 1.05) or “low metabolizers” (C0/D ≥ 1.05). Subsequently, several studies have shown that high-metabolizer KTRs have poorer allograft function and even a decrease in allograft survival.^10^, ^11^, ^12^ However, it is unclear if this is linked to higher CNI-allograft toxicity^13^ or to a higher rate of biopsy-proven acute rejection (BPAR) history in these patients.^14^

Importantly, the characterization of patients’ tacrolimus metabolic status using C0/D can be difficult to assess in a real-life setting. First, reproducibility of the C0/D ratio is uncertain, especially during the first months posttransplantation. Second, the optimal time to classify a patient as a high metabolizer with this tool is yet to be determined. Indeed, most studies investigated C0/D as a time-dependent variable, allowing a correlation between allograft survival and the time spent with a C0/D < 1.05 but did not provide an indication of its clinical application.^13^, ^14^, ^15^, ^16^ However, early and accurate identification of these patients is important to implement immunosuppression modulation.

The purpose of our study was to determine the reproducibility of the C0/D ratio after kidney transplantation to define the optimal and earliest time to consider patients as high metabolizers. We then investigated allograft survival and posttransplantation outcomes in these patients, with a special focus on both BPAR and tacrolimus nephrotoxicity.

## Methods

### Study Population

All adult patients who received a kidney transplant between January 1, 2000, and December 31, 2019 at our institution, with a functional allograft at 1 month after transplantation, receiving either immediate-release or extended-release tacrolimus were included in the analysis. Simultaneous kidney-pancreas transplants, HIV-positive recipients, and patients undergoing LCP-tacrolimus therapy or without tacrolimus therapy were excluded.

C0/D was defined as the ratio of blood tacrolimus trough level (ng/ml) to the daily total tacrolimus dose (mg). C0 was measured 12 or 24 hours after drug intake, depending on the tacrolimus formulation (immediate-release tacrolimus and extended-release tacrolimus, respectively). High tacrolimus metabolizer patients (“High”) were defined by history of C0/D < 1.05 on more than 75% of previous measurements at a defined timepoint. Low tacrolimus metabolizers (“Low”) were defined by history of C0/D ≥ 1.05 on more than 75% of previous dosages at a defined timepoint. Patients not corresponding to either High or Low were considered as “Variable.” The intraindividual coefficient of tacrolimus variability was determined using the following formula, as previously published^3^: CV = [((X_mean_− X_1_) + (X_mean_− X_2_) + …. + (X_mean_− X_n_))/n]/ X_mean_ × 100

### Available Data

Recipient characteristics included age; gender; body mass index; transplantation rank; preemptive status; use of machine perfusion; as well as history of diabetes, hypertension, and cardiac disease. Donor features included age, gender, and donor type (living or deceased from extended criteria donor or standard criteria donor). Baseline transplantation parameters included cold ischemia time, number of human leukocyte antigen A-B-DR incompatibilities, induction therapy, and the occurrence of delayed graft function defined by the necessity for dialysis during the first postoperative week. Posttransplantation parameters included the occurrence of BPAR, *de novo* donor-specific antibodies (dnDSAs), infectious complications (BK polyomavirus [BKV], cytomegalovirus [CMV], and severe bacterial infection), and estimated glomerular filtration rate measurements. BPAR was defined according to the 2019 Banff classification on systematic and/or for cause allograft biopsies.^17^ Occurrence of dnDSAs was considered significant when mean fluorescence index was ≥ 2000 (Luminex). CMV infection was defined by CMV viremia (primary infection or reactivation). BKV infection was defined as the occurrence of BKV viremia and/or BKV nephropathy. Severe bacterial infection was defined by a bacterial infection episode requiring hospitalization. The follow-up and the collection of data stopped upon return to dialysis, retransplantation, or death, whichever occurred first.

### Ethics Statement

Following informed consent, all patients’ data were extracted from the DIVAT database (Données Informatisées et VAlidées en Transplantation; www.divat.fr, approved by the Comité National de l’Informatique et des Libertés CNIL, No.914184) and deidentified to respect confidentiality. The clinical and research activities being reported are consistent with the Principles of the Declaration of Istanbul as outlined in the “Declaration of Istanbul on Organ Trafficking and Transplant Tourism.”

### Study Outcomes

The first outcome studied was the determination of the earliest accurate time for the categorization of High patients by investigating the variability of C0/D during the first 6 months posttransplantation. Subsequent studies included posttransplantation outcomes such as patient and allograft survival, occurrence of a first rejection episode, occurrence of dnDSA, posttransplant diabetes mellitus, infectious complications, and allograft function. These were investigated from the earliest defined time point after transplantation, which accurately defined the patients as High, Variable, and Low. Finally, the severity of chronic and CNI-related injuries, as defined by the Banff classification (cg, ct, ci, cv, and ah), was investigated 3 and 12 months posttransplantation in patients who underwent both biopsy assessments (either for cause or protocol).

### Statistical Analyses

Using the above definition for High and Low patients, we assessed the variability in groups during the first 6 months posttransplantation by investigating the percentage of patients being High at T = X months (where, X = 1–5 months after transplantation) and remaining in the same group at T∗ = 6 months. The Brier score was determined for evaluation of the predictability of patient’s status from T = X months and T = 6 months.^18^ The earliest and accurate timepoint for categorization was then used to investigate allograft outcomes. We completed our study by evaluating the relationship between the patient’s status and both death-censored graft survival (defined as the time between T = X months and the first event between return to dialysis, preemptive retransplantation) and patient survival, occurrence of BPAR, dnDSA, infectious complications, and posttransplant diabetes mellitus. To compare the outcomes and consider possible confounders, cause-specific Cox models were used for time-to-event.^19^ Variables significantly associated with the outcome in univariate regressions were retained (*P* < 0.20) in the multivariable models. The Benjamini and Hochberg method was used to adjust the level of significance and take into account multiple hypothesis testing.^20^ The log-linearity assumption was automatically checked; rejection of this assumption occurred when the Bayesian Information Criterion decreased using natural spline transformation compared with the inclusion of the covariate in its natural scale. In case of violations, the variables were categorized. Hazard proportionality was checked by plotting log-minus-log survival curves according to the 3 groups of interest and studying the Schoenfeld residuals.^21^ The adjusted survival curves were obtained by weighing the individual contributions by the inverse of the probability of being in the group and compared using the log-rank test for adjusted survival curves.^22^^,^^23^ Patients with missing data on the covariates retained for the multivariable models were excluded from the corresponding analysis. All analyses were performed using the RStudio software v 4.0.3 (R Foundation for Statistical Computing, Vienna, Austria).

## Results

### Description of Cohort and Reproducibility of C0/D Assessment

We included 1979 KTRs who were alive with a functional allograft 1 month after transplantation and receiving either immediate-release or extended-release tacrolimus. During follow-up, 171 return-to-dialysis and 179 deaths with a functioning graft were observed (Supplementary Figure S1).

**Figure 1:**
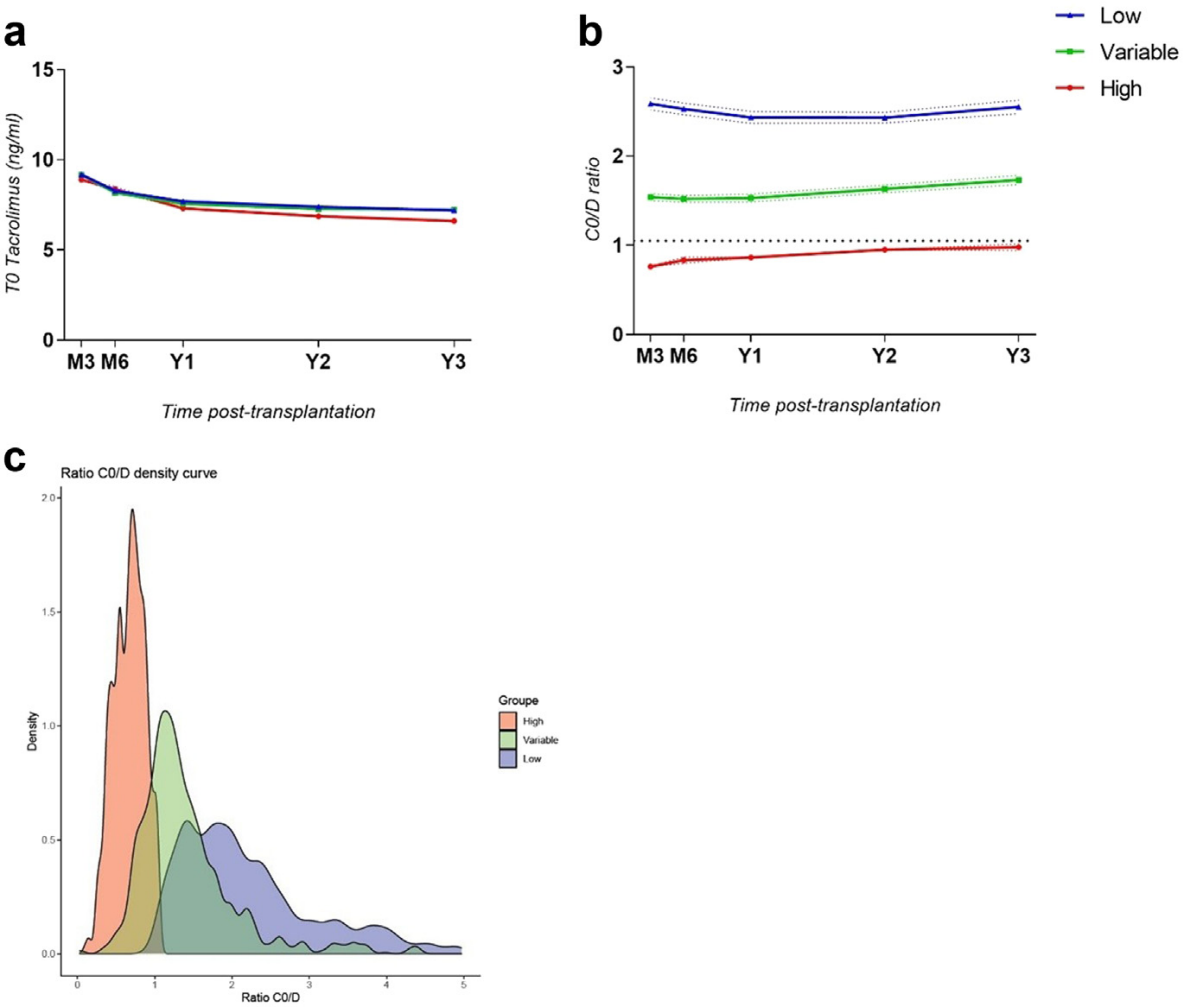
(a) Evaluation of tacrolimus trough level among groups defined from T = 2-months during the first 3 years posttransplantation. (b) Evaluation of C0/D ratio among groups defined from T = 2 months during the first 3 years posttransplantation. (c) Density plot of all measured C0/D values among groups defined from the second month posttransplantation. C0/D, tacrolimus trough-level / total daily dose.

At T = 1 month posttransplantation, 752 KTRs (38%) were High (> 75% of previous C0/D measurements < 1.05), of which 61% were still considered High at T = 6 months. At T = 2 months, 499 KTRs (25%) were High, 84% of which were considered High at T = 6 months (Table 1). Thereafter, at the following times T = 3 to 5 months, the percentages of patients still considered High at T = 6 months were 84.5%, 89%, and 95%, respectively. The complete evolution of tacrolimus C0 and C0/D during the first years posttransplantation in the different groups considered from T = 2 months is presented in Figure 1a and b. Despite very similar trough levels between groups over time, the mean C0/D remained < 1.05 in High patients, during the complete follow-up. The density plot of all C0/D values among groups, defined at the second month posttransplantation, confirmed good reproducibility over time from this time point (Figure 1c). Finally, the Brier score for determining high patients at 2 months was 0.06, which was better than for determining High patients at 1 month (0.106).

**Table 1:** Representation of the number of patients that changed group between M∗ and M6 when defined at 1–3 months posttransplantation

Time of assessment	Categorization at M∗ posttransplantation	Average number of C0/D used	Categorization at M6 posttransplantation, *n (%)*		
			High	Variable	Low
M1 posttransplantation	High	1.87	458 (60.9%)	281 (37.4%)	13 (1.7%)
	Variable	2.00	31 (8.1%)	180 (47.0%)	172 (44.9%)
	Low	1.86	2 (0.002%)	87 (10.5%)	755 (89.5%)
M2 posttransplantation	High	3.69	420 (84.2%)	79 (15.8%)	0 (0%)
	Variable	3.80	71 (9.8%)	440 (60.5%)	216 (29.7%)
	Low	3.66	0 (0%)	29 (3.8%)	724 (96.2%)
M3 posttransplantation	High	4.62	475 (84.5%)	87 (15.4%)	0 (0%)
	Variable	4.44	16 (3.4%)	407 (87.2%)	44 (9.4%)
	Low	4.61	0 (0%)	54 (5.7%)	896 (94.3%)

C0/D, tacrolimus trough level/dose; M, month.

Among the patients defined as “High” at 2 months posttransplantation, 420 (84.2%) of them remained “High” at 6 months, 79 (15.8%) were defined as “Variable” at 6 months posttransplantation, and 0 (0%) were “Low” at 6 months.

At T = 2 months, High patients were younger than Variable and Low patients (aged 48 vs. 52 and 55 years, respectively) and received an allograft from a standard criteria donor more often (52.7% vs. 45.1 and 41.6%, respectively). Nevertheless, High patients had a significantly lower estimated glomerular filtration rate at 2 months than Variable and Low patients (46.1 ml/min vs. 48.5 and 50.0 ml/min, respectively). This difference was possibly related to both a higher history of rejection episodes during the first 2 months (6% vs. 4.3 and 3%, respectively) and higher CNI total dose (14.3 mg/d vs. 7.6 and 4.6 mg/d, respectively). Directly linked to the groups’ definition, C0/D was lower in High patients than in Variable and Low (0.66 vs. 1.39 and 2.55, respectively). Finally, the tacrolimus CV calculated during the first year posttransplantation was lower in High patients than in Low and Variable patients (27.1 vs. 30.8 vs 32.3, respectively). The complete characteristics of the patients at T = 2 months are described in Table 2.

**Table 2:** Description of the 1979 patients with a functioning graft at 2 months posttransplantation according to their fast-metabolizer status

	Whole sample (*N* = 1979)			High patients (*n* = 499)			Variable patients (*n* = 727)			Low patients (*n* = 753)			*P*-value
	NA	*n*	%	NA	*n*	%	NA	*n*	%	NA	*n*	%	
Male recipient	0	1241	62.7	0	300	60.1	0	457	62.7	0	484	64.3	0.3282
Retransplantation	0	509	25.7	0	121	24.2	0	189	26.0	0	199	26.4	0.6729
Preemptive	0	379	19.1	0	97	19.4	0	143	19.7	0	139	18.5	0.8246
History of diabetes	0	342	17.3	0	80	16.0	0	118	16.2	0	144	19.1	0.2354
History of dyslipidemia	0	917	46.3	0	203	40.7	0	338	46.5	0	376	49.9	0.0057
History of hypertension	0	1796	90.7	0	443	88.8	0	663	91.2	0	690	91.6	0.2033
History of cardiac disease	0	611	30.9	0	133	26.6	0	216	29.7	0	262	34.8	0.0066
Male donor		1153	58.3		298	59.7		430	59.1		425	56.4	0.4280
Donor type	0			0			0			0			
Living		328	16.6		90	18.0		124	17.1		114	15.1	0.3652
SCD		904	45.7		263	52.7		328	45.1		313	41.6	0.0005
ECD		747	37.7		146	29.2		275	37.8		326	43.3	<0.0001
HLA-A-B-DR incompatibilities > 4	0	374	18.9	0	108	21.6	0	129	17.7	0	137	18.2	0.1893
Depleting induction	0	999	50.5	0	299	59.9	0	366	50.3	0	334	44.4	<0.0001
Machine perfusion	185	479	26.7	31	143	30.5	53	177	26.3	101	159	24.4	0.0671
Delayed graft function	24	624	31.9	8	175	35.6	10	216	30.1	6	233	31.2	0.1122
BPAR in the first 2 mos	0	84	4.2	0	30	6.0	0	31	4.3	0	23	3.0	0.0396
Tacrolimus at 2 mos	1			1			0			0			
Extended release		736	37.2		260	52.2		284	39.0		192	25.5	<0.0001
Immediate release		1242	62.7		238	47.8		443	61.0		561	75.5	< 0.0001


	NA	m	SD	NA	m	SD	NA	m	SD	NA	m	SD	*P*-value
Recipient age (yrs)	0	52.0	14.5	0	48	14.3	0	51.8	14.7	0	54.9	13.8	< 0.0001
Recipient BMI (kg/m^2^)	0	24.5	4.4	0	24.2	4.5	0	24.4	4.2	0	24.8	4.4	0.0414
Donor age (yrs)	1	53.3	15.9	0	50.4	15.6	1	53.3	16.1	0	55.2	15.7	< 0.0001
Donor creatinine (ml/min/m^2^)	4	86.8	49.2	2	89.9	54.8	0	86.5	48.0	2	85.0	46.2	0.2235
Cold ischemia time (h)	0	15.8	9.9	0	13.9	8.8	0	15.5	9.9	0	17.3	10.4	< 0.0001
eGFR at 2 mo (ml/min/m^2^)	162	48.4	19.4	49	46.1	18.9	41	48.5	19.9	72	50.0	18.9	0.0014
Tac dose (2 mos, mg/d)	0	8.1	4.9	0	14.3	4.9	0	7.6	2.4	0	4.6	1.7	< 0.0001
Tac trough level (2 mos, ng/ml)	0	9.6	3.3	0	8.8	2.3	0	9.8	3.7	0	9.8	3.4	< 0.0001
C0/D ratio at 2 mos	0	1.65	1.41	0	0.66	0.20	0	1.39	0.70	0	2.55	1.79	< 0.0001
Tacrolimus CV during first yr (%)	0	30.4	16.8	0	27.1	14.9	0	32.3	15.6	0	30.8	18.8	< 0.0001

BMI, body mass index; BPAR, biopsy-proven acute rejection; C0/D, tacrolimus trough level/dose; ECD, expanded criteria donor; eGFR, estimated glomerular filtration rate; HLA, human leukocyte antigen; NA, not available (missing); SCD, standard criteria donor; Tac, tacrolimus.

*P*-values are obtained using Chi-square test for categorical variables and ANOVA for continuous variables.

### Patient and Allograft Survival in High Patients Defined at T = 2 Months

From T = 2 months, we estimated the death-censored graft survival and patient survival at 5 and 10 years posttransplantation in the different groups (High, Variable, and Low), after adjusting for confounding factors differentially expressed among the groups (age, tacrolimus formulation, history of rejection during the first 2 months, type of donor, etc.).

**Figure 2:**
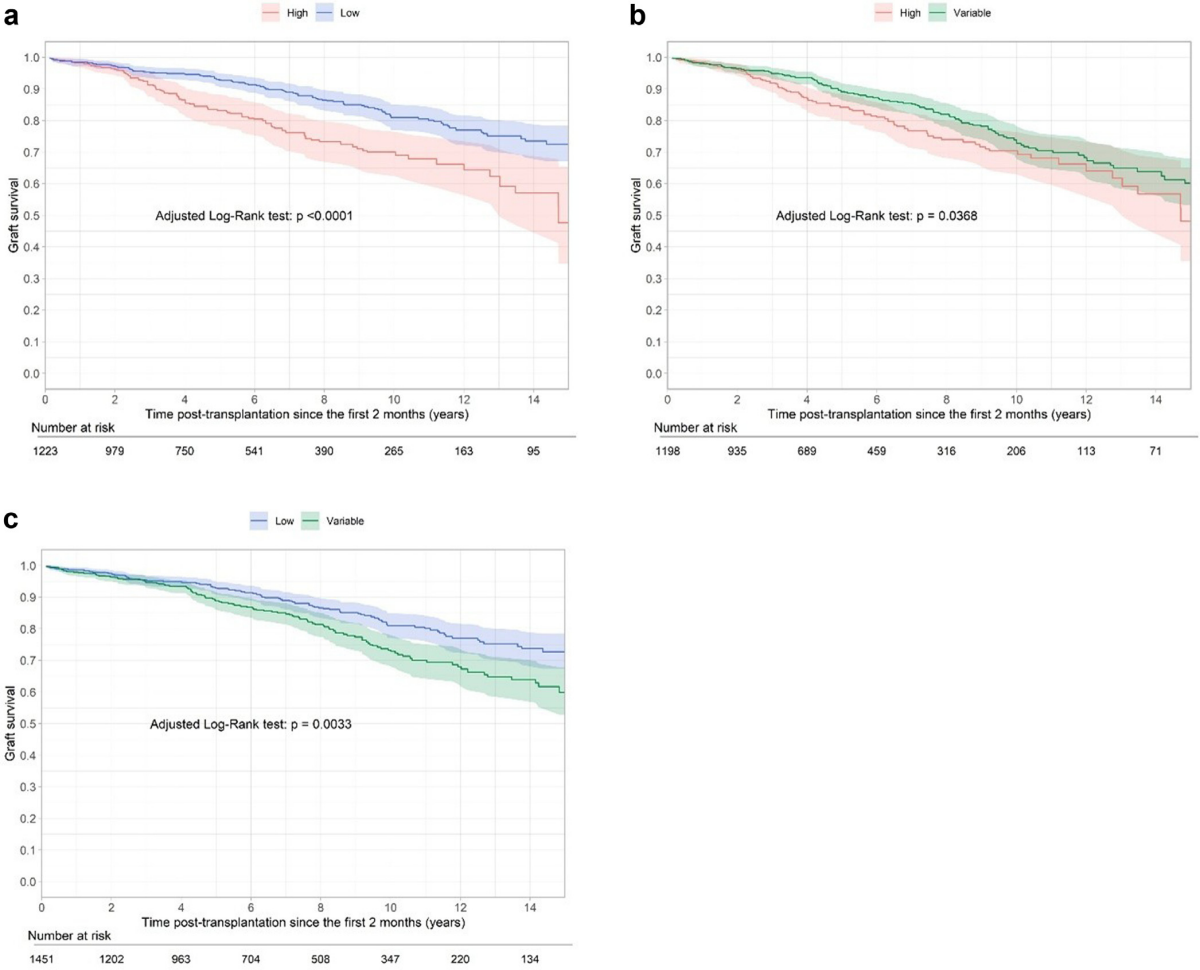
(a) Long-term confounder-adjusted graft survival from T = 2 months posttransplantation according to the patient’s status (High vs. Low) estimated from the weighted Kaplan-Meier estimator. (b) Long-term confounder-adjusted graft survival from T = 2 months posttransplantation according to the patient’s status (High vs. Variable) estimated from the weighted Kaplan-Meier estimator. (c) Long-term confounder-adjusted graft survival from T = 2 months posttransplantation according to the patient’s status (Variable vs. Low) estimated from the weighted Kaplan-Meier estimator.

The confounder-adjusted death-censored graft survival rates at 5 and 10 years posttransplantation were 83% and 70% for High patients, 89% and 73% for Variable patients, and 93% and 81% for Low patients, respectively(*P* < 0.0001, Figure 2). Consequently, the adjusted risk of death-censored allograft loss was significantly higher in High patients (CS-HR = 2.00, 95% CI = 1.48–2.69) and Variable patients (CS-HR = 1.51, 95% CI = 1.17–1.97*, P* < 0.0001) than in Low patients (Table 3).

**Table 3:** Results of the univariate and multivariate cox model (*n* = 1936) studying the risk of graft failure from the second month posttransplantation

	Univariate Analysis			Multivariate analysis			
	CS-HR	95% CI	*P*-value	CS-HR	95% CI	*P*-value	Adjusted *P*-value
Metabolizer status (ref: low)			0.0119			< 0.0001	< 0.0001
High	1.87	[1.41–2.47]		2.00	[1.48–2.69]		
Variable	1.39	[1.08–1.79]		1.51	[1.17–1.97]		
Retransplantation	1.57	[1.25–1.96]	<0.0001	1.54	[1.21–1.97]	0.0005	0.0016
Delayed graft function	1.85	[1.48–2.30]	< 0.0001	1.22	[0.96–1.56]	0.1029	0.1132
History of diabetes	1.48	[1.11–1.99]	0.0087	1.35	[0.99–1.84]	0.0593	0.0725
History of cardiac disease	1.59	[1.27–1.99]	< 0.0001	1.28	[1.01–1.64]	0.0428	0.0673
Donor type (ref: Living)			< 0.0001			< 0.0001	< 0.0001
SCD	1.83	[1.23–2.72]		1.16	[0.76–1.76]		
ECD	2.57	[1.71–3.84]		1.55	[1.00–2.41]		
Rejection episode in the first 2 mos posttransplantation	2.07	[1.40–3.07]	0.0003	1.99	[1.33–2.98]	0.0007	0.0016
Immediate release Tacrolimus	1.41	[1.05–1.90]	0.0223	1.71	[1.25–2.33]	0.0007	0.0016
MDRD	0.97	[0.97–0.98]	< 0.0001	0.98	[0.97–0.99]	< 0.0001	< 0.0001
Recipient age (yrs)	1.00	[0.99–1.01]	0.8103				
Male recipient	0.86	[0.69–1.07]	0.1715				
Recipient BMI (kg/m^2^)	0.99	[0.97–1.02]	0.4861				
Preemptive transplantation	0.67	[0.48–0.93]	0.0173				
Hypothermic machine perfusion	1.39	[0.98–1.97]	0.0625				
Cold ischemia time (h)	1.02	[1.01–1.03]	0.0017				
History of dyslipidemia	1.09	[0.87–1.36]	0.4456				
History of hypertension	0.91	[0.63–1.31]	0.6014				
Donor age (yrs)	1.01	[1.01–1.02]	0.0006				
Male donor	0.93	[0.75–1.16]	0.5178				
HLA-A-B-DR incompatibilities >4	1.16	[0.93–1.44]	0.1922				
Depleting induction	1.46	[1.17–1.81]	0.0007				

BMI, body mass index; CI, confidence interval; CS-HR, cause-specific hazard ratio; ECD, expanded criteria donor; HLA, human leukocyte antigen; MDRD, Modification of Diet in Renal Disease; SCD, standard criteria donor.

318 events observed during the follow-up; 43 patients were excluded because of missing data.

From T = 2 months, the confounder-adjusted risk of death did not significantly differ between the groups (CS-HR = 1.40, 95% CI = 1.00–1.93; and CS-HR = 1.01, 95% CI = 0.79–1.29 for High and Variable patients, respectively, compared to Low patients) (Supplementary Table S1).

### Allograft Rejection and dnDSA in High Patients Defined at T = 2 Months

**Figure 3:**
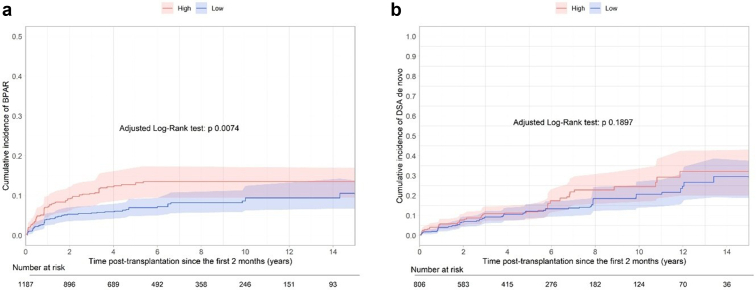
(a) Long-term confounder-adjusted death-censored occurrence of rejection from T = 2 months posttransplantation according to the patient’s status (High vs. Low) estimated from the weighted Kaplan-Meier estimator. (b) Long-term confounder-adjusted occurrence of de novo DSA from T = 2 months posttransplantation according to the patient’s status (High vs. Low) estimated from the weighted Kaplan-Meier estimator. DSA, donor-specific antibody.

From T = 2 months, the occurrence of allograft rejection was significantly higher in High patients (CS-HR = 1.71, 95% CI = 1.15–2.54) and Variable patients (CS-HR = 1.55, 95% CI = 1.07–2.54], *P* = 0.0151) than in Low patients after adjustment for confounding variables (Figure 3a, Table 4). Of note, patients with a history of rejection during the first 2 months were excluded from this analysis. Numerically, 268 patients presented with at least 1 rejection episode (89 High, 105 Variable, and 74 Low), of which 184 were diagnosed after the second month posttransplantation (59 High, 74 Variable, and 51 Low). High patients presented with a significantly higher occurrence of T-cell–mediated rejection and borderline lesions, whereas the occurrence of antibody-mediated rejection did not differ between the groups (Supplementary Figure S2). The severity of interstitial inflammation, tubulitis, and vasculitis according to the Banff classification did not differ among patients who presented with a T-cell–mediated rejection episode in the 3 groups (Supplementary Figure S3). In addition, the number of recurrent rejection episodes was similar (14.7% in High patients, 13.3% in Variable patients, and 13.0% in Low patients). In the multivariate analysis, the occurrence of dnDSA was not significantly higher in patients in the High group (CS-HR = 1.40, 95% CI= 0.96–2.05) (Figure 3b and Supplementary Table S2).

**Table 4:** Results of the univariate and multivariate cox model (*n* = 1881) studying the risk of allograft rejection from the second month posttransplantation

	Univariate Analysis			Multivariate Analysis			
	CS-HR	95% CI	*P*-value	CS-HR	95% CI	*P*-value	Adjusted *P*-value
Metabolizer status (ref: Low)			0.0011			0.0151	0.0219
High	2.01	[1.37–2.93]		1.71	[1.15–2.54]		
Variable	1.59	[1.11–2.27]		1.55	[1.07–2.22]		
Recipient age (yrs)	0.99	[0.98–1.00]	0.0743	0.98	[0.97–0.99]	0.0026	0.0101
History of diabetes	1.31	[0.91–1.90]	0.1512	1.50	[1.03–2.20]	0.0357	0.0498
Male donor	0.67	[0.50–0.89]	0.0066	0.65	[0.48–0.87]	0.0038	0.0103
HLA-A-B-DR incompatibilities > 4	1.47	[1.10–1.97]	0.0098	1.49	[1.11–2.01]	0.0079	0.0118
MDRD	0.99	[0.98–1.00]	0.0096	0.99	[0.98–1.00]	0.0043	0.0101
Retransplantation	0.97	[0.69–1.35]	0.8543				
Male recipient	1.04	[0.77–1.41]	0.8072				
Recipient BMI (kg/m^2^)	1.00	[0.97–1.04]	0.9087				
Preemptive transplantation	0.93	[0.64–1.37]	0.7266				
Hypothermic machine perfusion	1.23	[0.87–1.74]	0.2485				
Delayed graft function	1.20	[0.88–1.63]	0.2498				
Cold ischemia time (h)	1.00	[0.98–1.01]	0.5682				
History of dyslipidemia	1.23	[0.92–1.65]	0.1587				
History of hypertension	1.02	[0.61–1.70]	0.9465				
History of cardiac disease	1.19	[0.87–1.62]	0.2811				
Donor age (yrs)	1.00	[0.99–1.01]	0.9254				
Donor type (ref: Living)			0.4073				
SCD	1.00	[0.65–1.55]					
ECD	1.23	[0.79–1.90]					
Depleting induction	1.08	[0.81–1.45]	0.5932				
Immediate release Tacrolimus	0.75	[0.56–1.02]	0.0674				

BMI, body mass index; CI, confidence interval; CS-HR, cause-specific hazard ratio; ECD, expanded criteria donor; HLA, human leukocyte antigen; MDRD, modification of diet in renal disease; SCD, standard criteria donor.

179 events were observed during the follow-up. Patients with rejection episode during the first 2 months were excluded from the analysis; 98 patients were excluded because of missing data or occurrence of the outcome during the first two months.

### Infectious and Metabolic Complications in High Patients Defined at T = 2 Months

**Figure 4:**
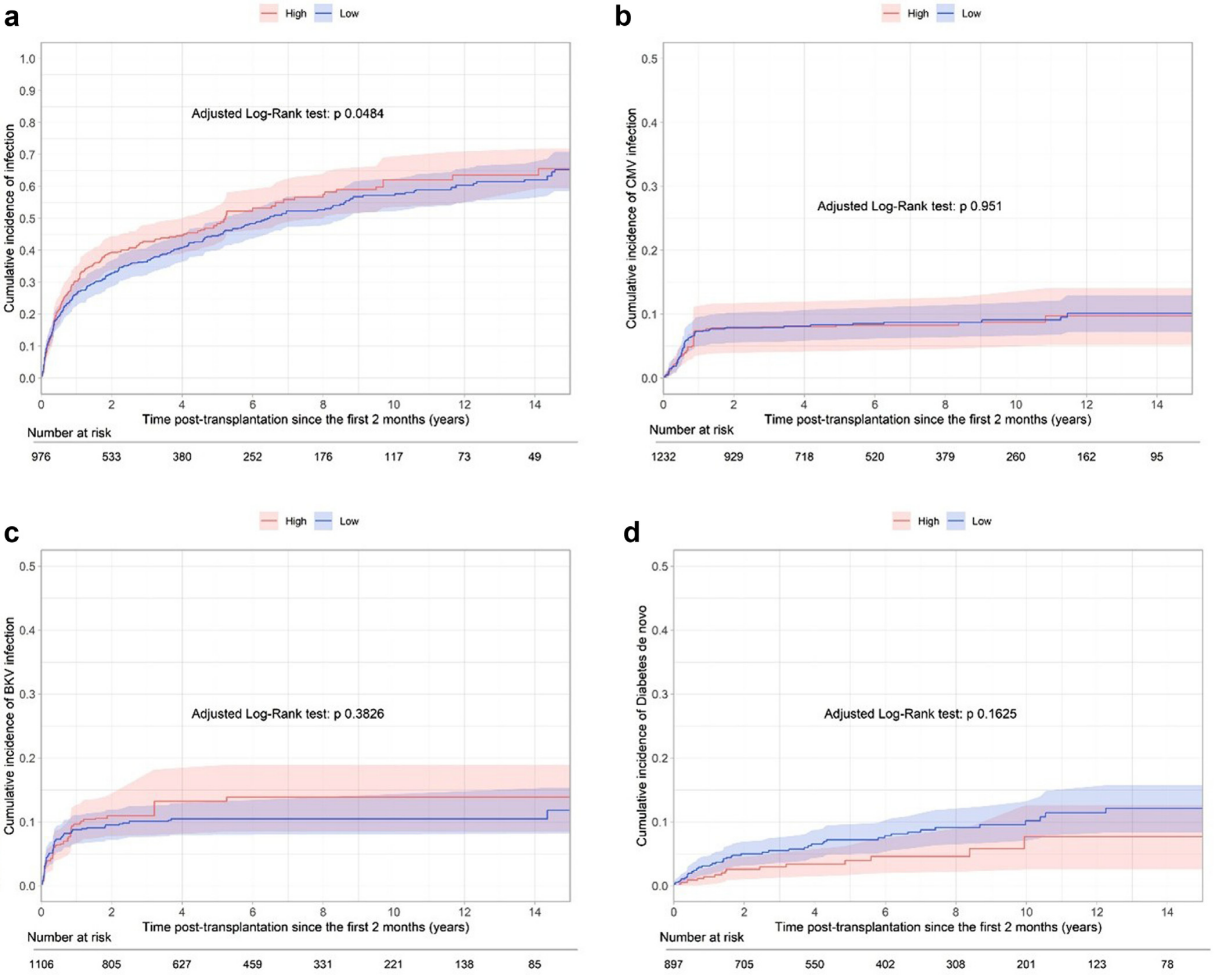
(a) Long-term confounder-adjusted occurrence of severe infectious complications from T = 2 months posttransplantation according to the patient’s status (High vs. Low) estimated from the weighted Kaplan-Meier estimator. (b) Long-term confounder-adjusted occurrence of CMV viremia from T = 2 months posttransplantation according to the patient’s status (High vs. Low) estimated from the weighted Kaplan-Meier estimator. (c) Long-term confounder-adjusted occurrence of BKV infection from T = 2 months posttransplantation according to the patient’s status (High vs. Low) estimated from the weighted Kaplan-Meier estimator. (d) Long-term confounder-adjusted occurrence of posttransplant diabetes mellitus from T = 2 months posttransplantation according to the patient’s status (High vs. Low) estimated from the weighted Kaplan-Meier estimator. BKV, BK polyomavirus; CMV, cytomegalovirus.

Severe infectious complications were more frequent in High patients than in Low patients (CS-HR = 1.27, 95% CI = 1.06–1.52, *P* = 0.0274) (Figure 4a and Supplementary Table S3). However, the risk for neither CMV nor BKV viremia was significantly increased in High patients (CS-HR = 0.96; 95% CI= 0.62–1.50; and CS-HR = 1.09,95% CI = 0.75–1.59), (Figure 4b and c and Supplementary Tables S4 and S5). Finally, the status from T=2 months had no impact on the observed occurrence of posttransplant diabetes mellitus in High and Variable patients compared with that in Low patients (CS-HR = 0.68, 95% CI = 0.39–1.20; and CS-HR = 0.74, 95% CI = 0.48–1.16) (Figure 4d and Supplementary Table S6).

### Allograft Function and Histological Injuries in Relation to High Status at T = 2 Months

**Figure 5:**
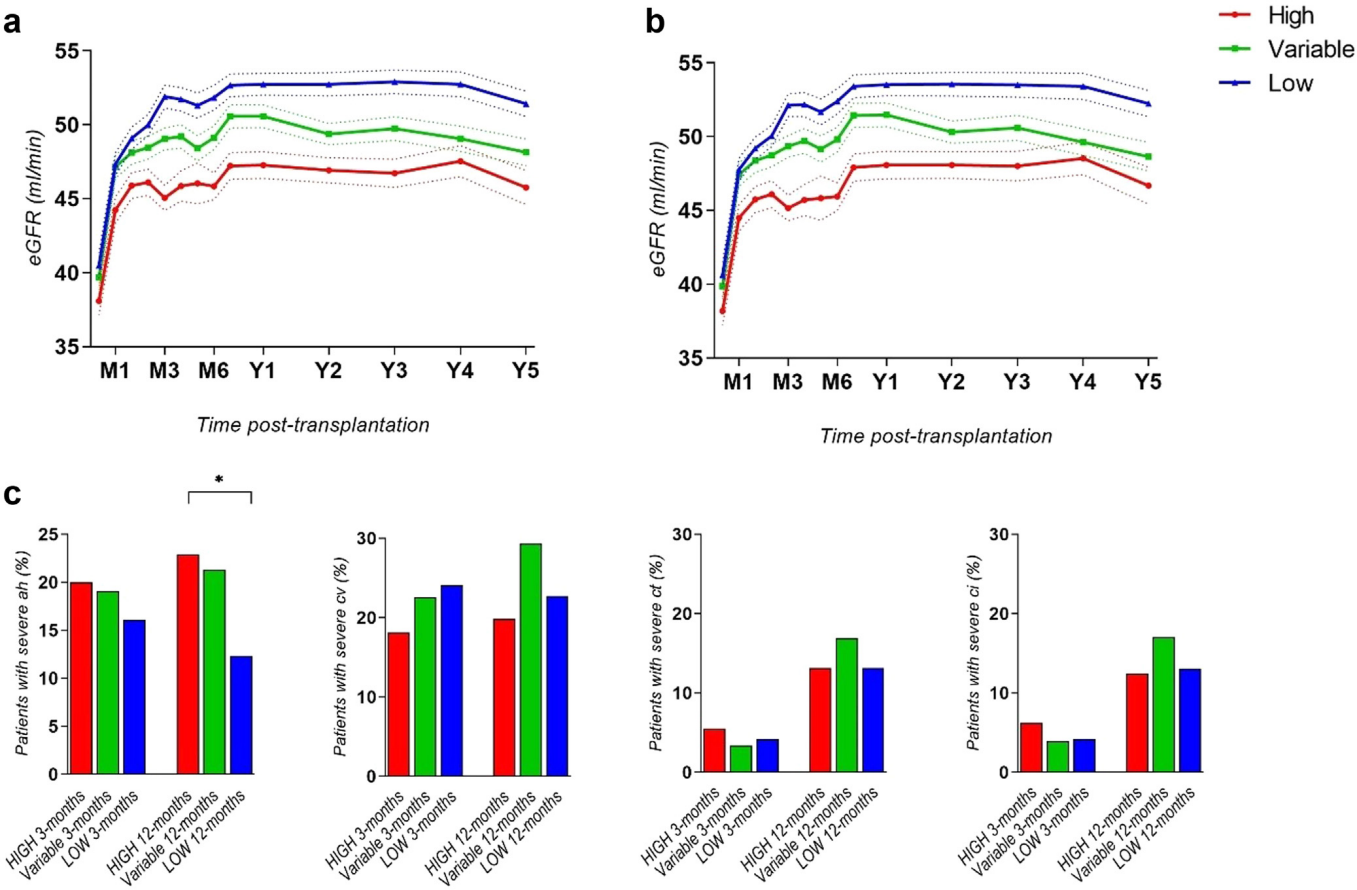
(a) Evaluation of allograft function estimated by eGFR (MDRD) among groups during the first years censored by allograft failure. *(*b*)* Evaluation of allograft function estimated by eGFR (MDRD) among groups during the first years censored by allograft failure and occurrence of a rejection episode. *(c)* Occurrence of severe chronic injuries in patients from High, Variable*,* and Low groups defined from the second month posttransplantation, in for cause and protocolar biopsies at 3 and 12 months posttransplantation. eGFR, estimated glomerular filtration rate; MDRD, modification of diet in renal disease.

We further evaluated the estimated glomerular filtration rate (censored by patient’s death and allograft loss) among the groups during the first years posttransplantation. We observed that estimated glomerular filtration rate at 3 years was significantly lower in High patients than in Variable and Low patients (46.7 ml/min vs. 49.7 and 52.9 ml/min, respectively; *P*< 0.0001) (Figure 5a). Interestingly, when excluding patients who presented a rejection episode (to distinguish the specific effects of CNI toxicity), this difference remained broadly unchanged (48.0, ml/min vs. 50.6 and 53.5 ml/min in High, Variable, and Low patients respectively; *P* = 0.0002) (Figure 5b).

Finally, we assessed chronic histological injuries (according to the Banff classification) in available biopsies. The characteristics of the 469 patients with both third and 12th month biopsies included in this subgroup analysis (145 High, 146 Low, and 178 Variables) are presented in Supplementary Table S7. Importantly, severe arteriolar hyalinosis (ah ≥ 2) at 1 year was significantly higher in High patients than in Low patients (23.0% vs. 12.3%, *P* = 0.02) (Figure 5c). In addition, High patients presented with a similar rate of severe chronic tubular (ct), chronic interstitial (ci), and chronic vascular (cv) injury (i.e., Banff score ≥ 2) compared with Low patients. Overall, despite receiving fewer extended criteria donor allografts (27% vs. 47%), patients in the High group exhibited similar levels of chronic histological injuries and a higher degree of arteriolar hyalinosis as early as 1 year posttransplantation, underscoring the nephrotoxicity present in this group of patients.

## Discussion

Despite C0/D being described as a potential tool for identifying high tacrolimus metabolizers, the lack of data regarding its reproducibility and capacity to prospectively identify at-risk patients weakens its use in routine clinical practice. In our study, we showed that patients with a C0/D < 1.05 for at least 75% of the time during previous measurements can be accurately categorized as high metabolizers from the second month posttransplantation. Indeed, 85% of these remained high metabolizers at 6 months, and their mean C0/D remained < 1.05 during the first years posttransplantation. However, our definition of High patients, based on pragmatic clinical considerations, might be open to debate and considered subjective. Nevertheless, we believe that using a time-varying evaluation of the C0/D ratio to define High patients was particularly relevant in this study, because our specific aim was to determine the earliest time point after transplantation that could reliably identify those patients. Moreover, we demonstrated that our classification effectively categorizes patients for up to 3 years posttransplantation.

Importantly, High patients defined as soon as the second month, carried significantly worse outcomes than others, after adjustment on cofounding factors. Despite comparable C0, these patients had a higher risk of allograft rejection (mainly T-cell–mediated rejection), which was probably linked to absorption issues. We also showed that High patients had significantly worse allograft survival and function despite having a mean tacrolimus CV < 30%. This suggests that the CNI toxicity observed in high metabolizers was mainly related to high doses (and thus high metabolites) rather than to a high intraindividual variability.^24^^,^^25^ Analysis of chronicity scores in biopsies confirmed that despite receiving less extended criteria donors, the High group had a higher burden of chronic vascular injuries (especially arteriolar hyalinosis).^26^ Arteriolar hyalinosis is a pattern commonly observed in CNI toxicity^27^^,^^28^ and has recently been linked to patient’s survival in a large cohort of patients with chronic kidney disease.^29^ These histological injuries aligns with the poorer allograft function observed in these patients, even after censoring for the occurrence of rejection. Overall, this supports the conclusion of greater nephrotoxicity among patients in the High group, with a consequent impact on allograft function and survival, likely because of the prolonged time spent with a C0/D ratio < 1.05. Finally, contrary to previous reports, we did not observe a higher risk of BKV in High patients.^30^

The identification of a patients’ subgroup at risk of both allograft rejection (linked to a low exposure to immunosuppressive drugs) and CNI toxicity may initially seem counterintuitive. This is related to the different issues underlined by C0/D < 1.05, that is, fast metabolism and/or digestive absorption. Indeed, some of the High patients in our cohort were probably underexposed to CNI (because of digestive absorption issues) and thus probably not really “high tacrolimus metabolizers.”^31^, ^32^, ^33^ However, the majority of them required higher CNI doses to reach the target trough levels, leading to high tacrolimus peak levels, which are important initiators of nephrotoxicity.^34^^,^^35^ In addition, the local accumulation of tacrolimus metabolites (demonstrated to be highest among fast metabolizers) certainly contributes to the impairment of kidney function.^36^ The highest rate of infectious complications in High patients may be explained by this higher exposure to tacrolimus.

The percentage of high patients in our study was 25%, which is slightly higher than the reported incidence of CYP3A5 polymorphism in the European population.^37^^,^^38^ This is related to the inclusion in our definition of patients who both (i) are relatively low absorbers rather than fast metabolizers, and (ii) fast metabolizers due to genetic causes and non-genetic causes (mainly drug interactions). However, we acknowledge that data on CYP3A5 polymorphism (not routinely monitored in our cohort) and/or information on Afro-American ethnicity (which could not be collected because of French law) would have provided more granular and valuable insights into identifying at-risk patients.

Overall, our study shows that using the C0/D ratio as soon as the second month posttransplantation accurately identifies at-risk patients for whom the follow-up using only trough-level monitoring is insufficient. The measurement of tacrolimus AUC (not routinely performed in our center) may allow an individualized determination of tacrolimus exposure in this selected population.^39^ Other tools, such as TTV monitoring, may provide meaningful insights into this population, in which C0 alone does not seem to be well-correlated with immunosuppression load. In addition, High patients may benefit from an early conversion to nonnephrotoxic maintenance therapy (such as mTOR inhibitors or belatacept), especially in case of impaired allograft function.^40^, ^41^, ^42^, ^43^ Another strategy would be the use of LCP-tacrolimus, which might reduce nephrotoxicity by avoiding the CNI peak level.^44^^,^^45^ Further studies are required to establish the benefit of such strategies in this at-risk population of KTRs.

Our study has several limitations. The observational nature does not permit us to exclude possible unobserved confounders, such as socioeconomic status, which can influence patients’ adherence to their medications. In addition, the lack of measures of tacrolimus AUC prevents definitive conclusion regarding the real exposure to CNI among High patients. Genotype data regarding CYP3A5 expressers would have been of interest in accurately determining the causes of fast tacrolimus metabolism in the High group. We voluntarily decided not to investigate C0/D as a continuous time-dependent variable but rather on predefined threshold evaluated at different times posttransplantation. This was due to our objective, which was to determine an early and accurate timepoint after transplantation to identify High patients carrying the worst allograft outcomes. Finally, a prospective evaluation of an external cohort to validate our observations would be of great interest.

In conclusion, we report that the C0/D ratio is a pragmatic and simple tool that can be used in routine practice to identify patients at risk of allograft failure and rejection as soon as the second month posttransplantation. The altered exposure to tacrolimus in these patients suggest a need for immunosuppression tailoring, possibly through tacrolimus AUC or changes in drugs regiments, which will require further evaluation.

## Appendix

### List of Données Informatisées et VAlidées en Transplantation, (DIVAT) Cohort Collaborators (Medical Doctors, Surgeons, HLA Biologists)

Gilles Blancho, Julien Branchereau, Diego Cantarovich, Agnès Chapelet, Jacques Dantal, Florent Delbos, Clément Deltombe, Lucile Figueres, Charles Ronsin, Thibault Letellier, Clémence Petit, Claire Garandeau, Magali Giral, Caroline Gourraud-Vercel, Laurent Nicolet, Christine Kandel-Aznar, Ismaël Chelghaf, Clarisse Kerleau, Christophe Masset, Aurélie Meurette, Karine Renaudin, Simon Ville, and Alexandre Walencik.

## Disclosure

All the authors declared no competing interests.

## Supplementary Material

Supplementary File (PDF) **Figure S1.** Flowchart of the study. **Figure S2.** Incidence of rejection subgroups (T-cell–mediated rejection, antibody-mediated rejection, Borderline) among groups. **Figure S3.** Evolution of histological chronic injuries among groups. **Table S1.** Results of the univariate and multivariate model for the risk of death. **Table S2.** Results of the univariate and multivariate model for the risk of *de novo* DSA. **Table S3.** Results of the univariate and multivariate model for the risk of severe infection. **Table S4.** Results of the univariate and multivariate model for the risk of CMV viremia. **Table S5.** Results of the univariate and multivariate model for the risk of BKV viremia. **Table S6.** Results of the univariate and multivariate model for the risk of posttransplant diabetes. **Table S7.** Description of the sub cohort included for the histological analysis.
