# Supplementary material for: Early Determination of Tacrolimus Concentration–Dose Ratio Identifies Risk of Allograft Loss in Kidney Transplantation
Source: Kidney Int Rep. 2025 Feb 25;10(5):1428–40. doi: 10.1016/j.ekir.2025.02.014 (PMC12142798; doi:10.1016/j.ekir.2025.02.014)
Supplement: Supplementary File (PDF) — Figure S1. Flowchart of the study. Figure S2. Incidence of rejection subgroups (T-cell–mediated rejection, antibody-mediated rejection, Borderline) among groups. Figure S3. Evolution of histological chronic injuries among groups. Table S1. Results of the univariate and multivariate model for the risk of death. Table S2. Results of the univariate and multivariate model for the risk of de novo DSA. Table S3. Results of the univariate and multivariate model for the risk of severe infection. Table S4. Results of the univariate and multivariate model for the risk of CMV viremia. Table S5. Results of the univariate and multivariate model for the risk of BKV viremia. Table S6. Results of the univariate and multivariate model for the risk of posttransplant diabetes. Table S7. Description of the sub cohort included for the histological analysis. [file mmc1.pdf]

Supplementary Material

Figure S1. Flowchart of the study (word file)
Figure S2. Incidence of rejection subgroups (TCMR, ABMR, Borderline) among groups (word file)
Figure S3. Evolution of histological chronic injuries among groups (word file)
Table S1. Results of the univariate and multivariate model for the risk of death (word file)
Table S2. Results of the univariate and multivariate model for the risk of de novo DSA (word file)
Table S3. Results of the univariate and multivariate model for the risk of severe infection (word file)
Table S4. Results of the univariate and multivariate model for the risk of CMV viremia (word file)
Table S5. Results of the univariate and multivariate model for the risk of BKV viremia (word file)
Table S6. Results of the univariate and multivariate model for the risk of post-transplant diabetes (word file)
Table S7. Description of the sub cohort included for the histological analysis (word file)

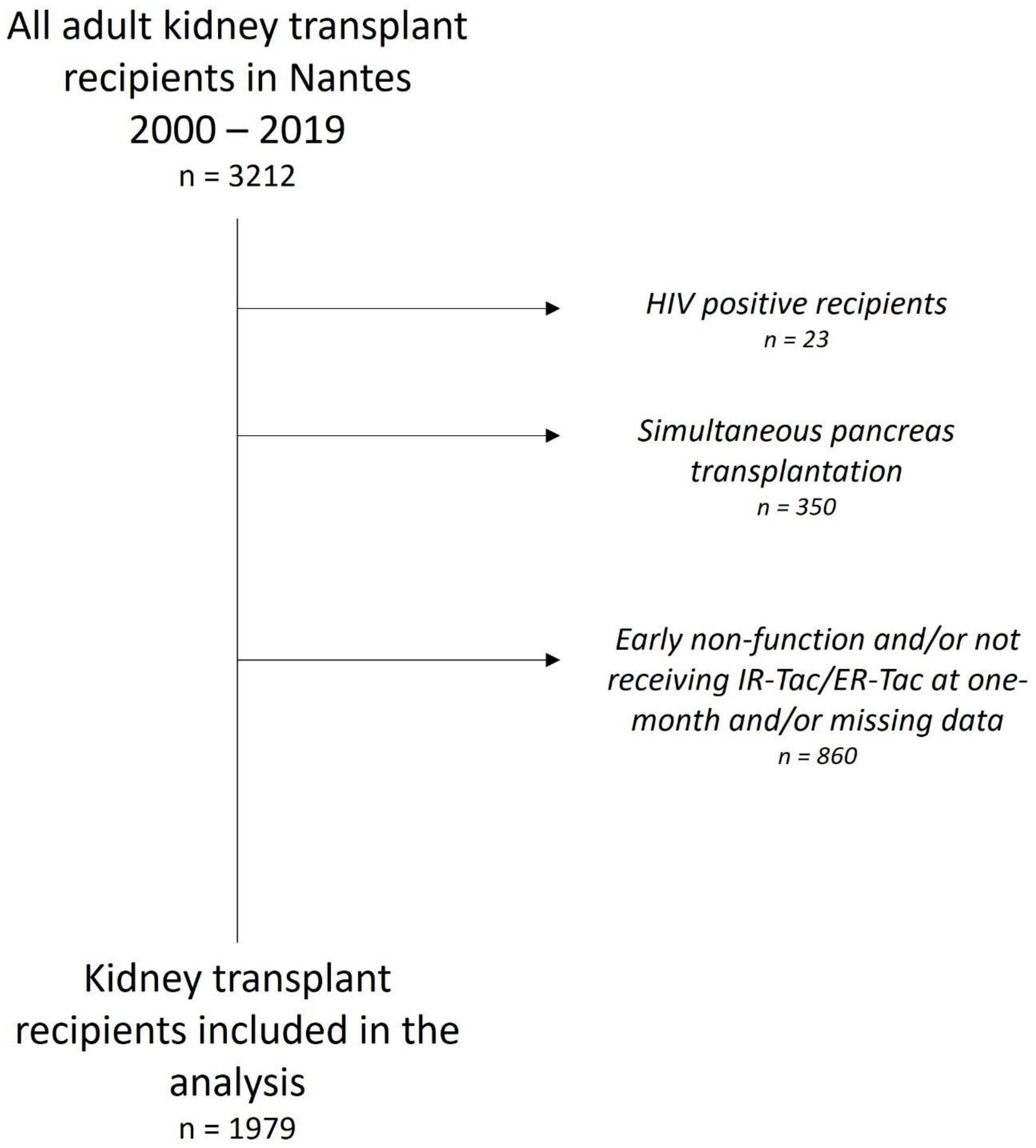

Figure S1. *Flow chart of the study*

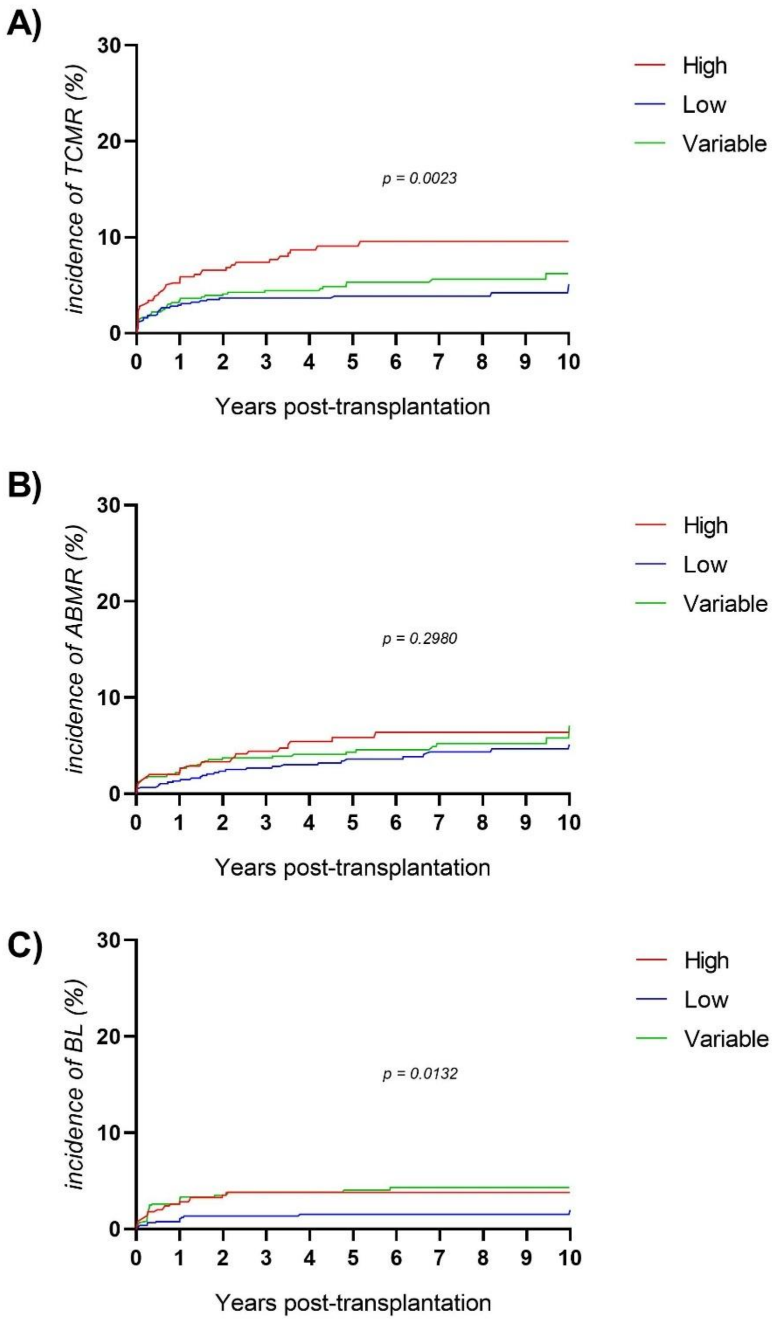

Figure S2. *Panel A. Incidence of TCMR among groups (censored at 10 years post-transplantation). Panel B. Incidence of ABMR among groups (censored at 10 years post-transplantation). Panel C. Incidence of Borderline Lesions among groups (censored at 10 years post-transplantation).*

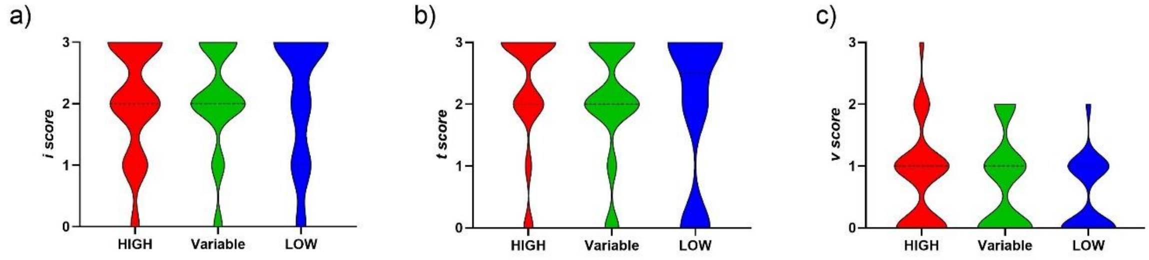

Figure S3. Histological severity of TCMR episodes represented by i (Panel A), t (Panel B) and v (Panel C) scores from the Banff Classification among HIGH, Variable and LOW patients.

Table S1. Results of the univariate and multivariate cox model (n = 1979) studying the risk of death (326 events observed during the follow-up)

	Univariate Analysis			Multivariate analysis			
	CS-HR	95% CI	p-value	CS-HR	95% CI	p-value	Adjusted p-value
**Metabolizer status** (ref : Normal)			0.0402			0.1116	0.1324
Fast	0.75	[0.55 ; 1.02]		1.40	[1.00 ; 1.93]		
Intermediate	0.75	[0.59 ; 0.96]		1.01	[0.79 ; 1.29]		
**Recipient age** (years)	1.08	[1.07 ; 1.09]	<0.0001	1.08	[1.07 ; 1.09]	<0.0001	<0.0001
**Recipient BMI** (kg/m²)	1.04	[1.02 ; 1.07]	0.0009	0.97	[0.95 ; 1.00]	0.0758	0.0867
**Preemptive transplantation**	0.51	[0.35 ; 0.73]	0.0003	0.62	[0.43 ; 0.90]	0.0129	0.0206
**History of diabetes**	2.97	[2.32 ; 3.79]	<0.0001	2.12	[1.64 ; 2.75]	<0.0001	<0.0001
**History of cardiac disease**	2.49	[2.01 ; 3.10]	<0.0001	1.77	[1.42 ; 2.21]	<0.0001	<0.0001
**Depleting induction**	1.22	[0.98 ; 1.52]	0.0703	1.43	[1.14 ; 1.80]	0.0023	0.0046
**Male recipient**	1.20	[0.95 ; 1.51]	0.1250				
**Hypothermic machine perfusion**	2.65	[1.97 ; 3.55]	<0.0001				
**Delayed graft function**	1.58	[1.27 ; 1.97]	<0.0001				
**Cold ischemia time** (hours)	1.01	[1.00 ; 1.02]	0.0080				
**Retransplantation**	1.09	[0.86 ; 1.39]	0.4620				
**History of dyslipidemia**	1.54	[1.24 ; 1.92]	0.0001				
**History of hypertension**	1.14	[0.76 ; 1.71]	0.5410				
**Donor age** (years)	1.06	[1.05 ; 1.06]	<0.0001				
**Male donor**	0.92	[0.73 ; 1.14]	0.4300				
**Donor type** (ref : Living)			<0.0001				
SCD	1.78	[1.09 ; 2.90]					
ECD	6.14	[3.83 ; 9.85]					
**HLA-A-B-DR incompatibilities > 4**	1.22	[0.98 ; 1.52]	0.0756				
**Rejection episode in the first 2 months post-transplantation**	0.81	[0.44 ; 1.48]	0.4910				
**Immediate release Tacrolimus**	0.70	[0.54 ; 0.90]	0.0056				
**MDRD**	0.98	[0.97 ; 0.99]	<0.0001				

CI, confidence interval; DGF, Delayed Graft Function; CS-HR : Cause specific hazard ratio; MDRD, Modification of Diet in Renal Disease.

Table S2. Results of the univariate and multivariate cox model (n = 1,286) studying the risk of de novo DSA occurrence (185 events observed during the follow-up, 693 patients were excluded due to missing data)

	Univariate Analysis			Multivariate analysis			
	**CS-HR**	**95% CI**	**p-value**	**CS-HR**	**95% CI**	**p-value**	**Adjusted p-value**
**Metabolizer status** (ref: Low)			0.0883			0.2038	0.2364
High	1.39	[1.02 ; 1.91]		1.40	[0.96 ; 2.05]		
Variable	1.26	[0.96 ; 1.65]		1.24	[0.88 ; 1.76]		
**Re-transplantation**	1.46	[1.13 ; 1.88]	0.0037	1.77	[1.22 ; 2.56]	0.0024	0.0047
**Recipient age** (years)	0.99	[0.98 ; 1.00]	0.0035	0.98	[0.97 ; 0.99]	0.0022	0.0046
**History of cardiac disease**	1.53	[1.19 ; 1.97]	0.0009	1.33	[0.95 ; 1.85]	0.0958	0.1278
**HLA-A-B-DR incompatibilities > 4**	1.52	[1.20 ; 1.93]	0.0006	1.92	[1.42 ; 2.59]	<0.0001	0.0001
**Pre-transplantation DSA**	4.52	[1.08 ; 1.74]	<0.0001	3.80	[2.38 ; 6.07]	<0.0001	<0.0001
**Rejection episode in the first 2 months post-transplantation**	1.78	[1.04 ; 3.05]	0.0356	0.96	[0.43 ; 2.18]	0.9310	0.9550
**Cold ischemia time** (hours)	1.00	[0.99 ; 1.01]	0.9554				
**Male recipient**	1.11	[0.86 ; 1.42]	0.4185				
**Recipient BMI** (kg/m^2)	0.99	[0.96 ; 1.02]	0.4112				
**Pre-emptive transplantation**	1.00	[0.74 ; 1.36]	0.9873				
**Hypothermic machine perfusion**	1.11	[0.79 ; 1.55]	0.5633				
**Delayed graft function**	1.06	[0.82 ; 1.37]	0.6766				
**History of diabetes**	1.29	[0.93 ; 1.79]	0.1322				
**History of dyslipidemia**	0.91	[0.72 ; 1.16]	0.4556				
**History of hypertension**	0.87	[0.58 ; 1.31]	0.5141				
**Donor age** (years)	1.00	[0.99 ; 1.01]	0.4398				
**Male donor**	1.04	[0.81 ; 1.32]	0.7832				
**Donor type** (ref: Living)			0.2004				
SCD	1.36	[0.96 ; 1.92]					
ECD	1.17	[0.80 ; 1.71]					
**Depleting induction at transplantation**	1.37	[1.08 ; 1.74]	0.0101				
**Immediate release Tacrolimus**	0.73	[0.56 ; 0.96]	0.0223				
**MDRD**	1.00	[0.99 ; 1.01]	0.6044				

CI, confidence interval; DGF, Delayed Graft Function; CS-HR : Cause specific hazard ratio; MDRD, Modification of Diet in Renal Disease.

Table S3. Results of the multivariate cox model (n = 1,725) studying the risk of severe infection (857 events observed during the follow-up, 254 patients were excluded due to missing data)

	Univariate Analysis			Multivariate analysis			
	CS-HR	95% CI	p-value	CS-HR	95% CI	p-value	Adjusted p-value
**Metabolizer status** (ref: Low)			0.2083			0.0274	0.0382
High	1.18	[0.99 ; 1.40]		1.27	[1.06 ; 1.52]		
Variable	1.10	[0.94 ; 1.29]		1.14	[0.97 ; 1.33]		
**Recipient age** (years)	1.02	[1.01 ; 1.02]	<0.0001	1.01	[1.00 ; 1.02]	0.0265	0.0376
**Male recipient**	0.75	[0.66 ; 0.86]	<0.0001	0.76	[0.66 ; 0.88]	0.0001	0.0007
**Male donor**	0.85	[0.74 ; 0.97]	0.0186	0.86	[0.75 ; 0.99]	0.0301	0.0398
**Donor type** (ref: Living)			<0.0001			0.0004	0.0012
SCD	1.36	[1.10 ; 1.69]		1.31	[1.05 ; 1.64]		
ECD	2.21	[1.78 ; 2.74]		1.84	[1.44 ; 2.35]		
**History of diabetes**	1.47	[1.24 ; 1.74]	<0.0001	1.30	[1.09 ; 1.55]	0.0038	0.0125
**History of cardiac disease**	1.23	[1.07 ; 1.42]	0.0047	1.12	[0.96 ; 1.30]	0.1397	0.1436
**Depleting induction**	1.28	[1.12 ; 1.47]	0.0003	1.22	[1.06 ; 1.40]	0.0057	0.0142
**Re-transplantation**	1.13	[0.97 ; 1.32]	0.1065				
**Recipient BMI** (kg/m²)	1.01	[0.99 ; 1.02]	0.2942				
**Pre-emptive transplantation**	0.80	[0.67 ; 0.96]	0.0178				
**Hypothermic machine perfusion**	1.52	[1.29 ; 1.78]	<0.0001				
**Delayed graft function**	1.18	[1.03 ; 1.37]	0.0193				
**Cold ischemia time** (hours)	1.01	[1.00 ; 1.02]	0.0707				
**History of dyslipidemia**	1.24	[1.09 ; 1.42]	0.0016				
**History of hypertension**	1.04	[0.82 ; 1.31]	0.7570				
**Donor age** (years)	1.02	[1.01 ; 1.02]	<0.0001				
**HLA-A-B-DR incompatibilities > 4**	1.10	[0.96 ; 1.26]	0.1887				
**Rejection episode in the first 2 months post-transplantation**	1.16	[0.83 ; 1.61]	0.3890				
**Immediate release Tacrolimus**	0.82	[0.71 ; 0.95]	0.0074				
**MDRD**	0.99	[0.98 ; 1.00]	<0.0001				

CI, confidence interval; DGF, Delayed Graft Function CS-HR : Cause specific hazard ratio; MDRD, Modification of Diet in Renal Disease.

Table S4. Results of the univariate and multivariate cox model (n = 1,953) studying the risk of CMV viremia (152 events observed during the follow-up, 26 patients were excluded due to missing data)

	Univariate Analysis			Multivariate analysis			
	CS-HR	95% CI	p-value	CS-HR	95% CI	p-value	Adjusted p-value
**Metabolizer status** (ref: Low)			0.8048			0.9721	0.9827
High	1.16	[0.77 ; 1.75]		0.96	[0.62 ; 1.50]		
Variable	1.07	[0.74 ; 1.55]		0.96	[0.65 ; 1.40]		
**Re-transplantation**	0.69	[0.46 ; 1.03]	0.0711	0.62	[0.39 ; 0.98]	0.0386	0.0701
**Recipient age** (years)	1.02	[1.01 ; 1.04]	0.0001	1.01	[1.00 ; 1.03]	0.0418	0.0829
**Immediate release Tacrolimus**	0.47	[0.34 ; 0.65]	<0.0001	0.56	[0.40 ; 0.80]	0.0011	0.0079
**MDRD**	0.98	[0.97 ; 0.99]	0.0005	0.99	[0.98 ; 1.00]	0.0501	0.0986
**Depleting induction**	1.42	[1.03 ; 1.96]	0.0325	1.69	[1.17 ; 2.43]	0.0048	0.0169
**Male recipient**	0.89	[0.65 ; 1.23]	0.4912				
**Recipient BMI** (kg/m²)	1.02	[0.99 ; 1.06]	0.2185				
**Pre-emptive transplantation**	1.12	[0.75 ; 1.66]	0.5874				
**Hypothermic machine perfusion**	2.06	[1.47 ; 2.88]	<0.0001				
**Delayed graft function**	0.83	[0.58 ; 1.18]	0.2992				
**Cold ischemia time** (hours)	0.99	[0.97 ; 1.00]	0.0917				
**History of diabetes**	1.02	[0.66 ; 1.56]	0.9474				
**History of dyslipidemia**	1.32	[0.96 ; 1.81]	0.0904				
**History of hypertension**	0.79	[0.48 ; 1.31]	0.3671				
**History of cardiac disease**	0.90	[0.63 ; 1.29]	0.5661				
**Donor age** (years)	0.93	[1.01 ; 1.03]	<0.0001				
**Male donor**	0.85	[0.68 ; 1.28]	0.6662				
**Donor type** (ref: Living)			0.0033				
SCD	1.14	[0.68 ; 1.90]					
ECD	1.91	[1.15 ; 3.16]					
**HLA-A-B-DR incompatibilities >4**	1.23	[0.89 ; 1.69]	0.2084				
**Rejection episode in the first 2 months post-transplantation**	0.61	[0.23 ; 1.66]	0.3362				

CI, confidence interval; DGF, Delayed Graft Function; CS-HR : Cause specific hazard ratio; MDRD, Modification of Diet in Renal Disease.

Table S5. Results of the univariate and multivariate cox model (n = 1,769) studying the risk of BKV viremia (208 events observed during the follow-up, 210 patients were excluded due to missing data)

	Univariate Analysis			Multivariate analysis			
	CS-HR	95% CI	p-value	CS-HR	95% CI	p-value	Adjusted p-value
**Metabolizer status** (ref: Low)			0.2083			0.3450	0.3897
High	1.22	[0.86 ; 1.74]		1.09	[0.75 ; 1.59]		
Variable	1.34	[0.98 ; 1.83]		1.26	[0.92 ; 1.74]		
**Re-transplantation**	1.21	[0.91 ; 1.63]	0.1962	1.48	[1.01 ; 2.18]	0.0471	0.0791
**Recipient age** (years)	1.03	[1.02 ; 1.04]	<0.0001	1.02	[1.00 ; 1.03]	0.0079	0.0272
**Donor type** (ref: Living)			0.0002			0.0728	0.0991
SCD	1.39	[0.88 ; 2.20]		1.81	[0.98 ; 3.33]		
ECD	2.18	[1.39 ; 3.42]		1.95	[1.00 ; 3.79]		
**HLA-A-B-DR incompatibilities >4**	1.49	[1.14 ; 1.95]	0.0035	1.41	[1.05 ; 1.90]	0.0211	0.0481
**Depleting induction**	1.73	[1.32 ; 2.28]	<0.0001	1.40	[0.99 ; 1.99]	0.0575	0.0885
**Hypothermic machine perfusion**	2.29	[1.74 ; 3.02]	<0.0001	1.42	[1.00 ; 2.02]	0.0531	0.0882
**Cold ischemia time** (hours)	0.99	[0.97 ; 1.00]	0.0411	0.97	[0.95 ; 1.00]	0.0207	0.0472
**History of dyslipidemia**	1.57	[1.20 ; 2.05]	0.0011	1.27	[0.96 ; 1.69]	0.0953	0.1164
**Delayed graft function**	0.83	[0.62 ; 1.12]	0.2255				
**History of diabetes**	1.01	[0.71 ; 1.45]	0.9563				
**Male recipient**	1.21	[0.91 ; 1.61]	0.1854				
**Recipient BMI** (kg/m²)	1.01	[0.98 ; 1.04]	0.3871				
**Pre-emptive transplantation**	0.86	[0.60 ; 1.22]	0.3920				
**History of hypertension**	1.07	[0.67 ; 1.71]	0.7922				
**History of cardiac disease**	1.20	[0.91 ; 1.60]	0.2003				
**Donor age** (years)	1.02	[1.01 ; 1.03]	<0.0001				
**Male donor**	0.92	[0.71 ; 1.21]	0.5682				
**Rejection episode in the first 2 months post-transplantation**	1.02	[0.52 ; 1.98]	0.9643				
**Immediate release Tacrolimus**	0.67	[0.51 ; 0.88]	0.0039				
**MDRD**	0.99	[0.98 ; 1.00]	0.0231				

CI, confidence interval; DGF, Delayed Graft Function; CS-HR : Cause specific hazard ratio; MDRD, Modification of Diet in Renal Disease.

Table S6. Results of the univariate and multivariate cox model (n = 1,427) studying the risk of Post-Transplant Diabetes Mellitus (n = 98 events observed during the follow-up, 552 patients were excluded due to missing data).

	**Univariate Analysis**			**Multivariate analysis**			
	**CS-HR**	**95% CI**	**p-value**	**CS-HR**	**95% CI**	**p-value**	**Adjusted p-value**
**Metabolizer status** (ref: Low)			0.2656			0.2695	0.2883
High	0.66	[0.38 ; 1.16]		0.68	[0.39 ; 1.20]		
Variable	0.77	[0.50 ; 1.20]		0.74	[0.48 ; 1.16]		
**Donor type** (ref : Living)			0.0316			0.0332	0.0638
SCD	2.27	[1.12 ; 4.59]		2.27	[1.12 ; 4.59]		
ECD	2.39	[1.14 ; 5.00]		2.09	[1.00 ; 4.38]		
**Recipient BMI** (kg/m^2)	1.13	[1.08 ; 1.18]	<0.0001	1.12	[1.07 ; 1.17]	<0.0001	<0.0001
**History of hypertension**	5.05	[1.25 ; 20.5]	0.0234	4.68	[1.15 ; 19.0]	0.0311	0.0622
**Re-transplantation**	0.93	[0.59 ; 1.46]	0.7460				
**Recipient age** (years)	1.01	[1.00 ; 1.03]	0.1212				
**Male recipient**	0.92	[0.61 ; 1.38]	0.6843				
**Pre-emptive transplantation**	0.51	[0.26 ; 0.97]	0.0411				
**Hypothermic machine perfusion**	1.08	[0.61 ; 1.91]	0.7811				
**Delayed graft function**	1.33	[0.88 ; 2.01]	0.1722				
**Cold ischemia time** (hours)	1.02	[1.00 ; 1.04]	0.0239				
**History of dyslipidemia**	1.49	[1.00 ; 2.22]	0.0496				
**History of cardiac disease**	1.07	[0.68 ; 1.67]	0.7743				
**Donor age** (years)	1.00	[0.99 ; 1.01]	0.9865				
**Male donor**	1.00	[0.67 ; 1.49]	0.9842				
**HLA-A-B-DR incompatibilities >4**	1.00	[0.67 ; 1.51]	0.9920				
**Depleting induction**	0.77	[0.51 ; 1.16]	0.2160				
**Rejection episode in the first 2 months post-transplantation**	1.21	[0.45 ; 3.30]	0.7063				
**Immediate release Tacrolimus**	0.78	[0.50 ; 1.22]	0.2730				
**MDRD**	1.00	[0.99 ; 1.01]	0.9542				

CI, confidence interval; DGF, Delayed Graft Function; CS-HR : Cause specific hazard ratio; MDRD, Modification of Diet in Renal Disease.

Table S7. Description of the 469 patients with a functioning graft at 2-months post-transplantation according to their status included in the histological analysis (p-values are obtained using Chi-square test for categorical variables and using Student t-test for continuous variables).

	Whole sample (n=469)			High patients (n= 145)			Variable patients (n = 178)			Low patients (n=146)			p-value
	NA	n	%	NA	n	%	NA	n	%	NA	n	%	
**Re-transplantation**	0	69	14.7	0	25	17.2	0	27	15.1	0	17	11.6	0.3937
**Male recipient**	0	296	63.1	0	84	57.9	0	110	61.8	0	102	69.9	0.0972
**Pre-emptive**	0	110	23.4	0	39	26.9	0	38	21.3	0	33	22.6	0.4829
**Machine perfusion**	3	196	42.1	1	53	36.8	2	72	40.9	0	71	48.6	0.1157
**Delayed graft function**	2	117	25.0	0	40	27.6	2	41	23.3	0	36	24.6	0.6396
**History of diabetes**	0	95	20.2	0	23	15.8	0	37	20.7	0	35	24.0	0.2217
**History of dyslipidemia**	0	243	51.8	0	57	39.3	0	102	57.3	0	84	57.5	0.0014
**History of hypertension**	0	426	90.8	0	136	93.8	0	157	88.2	0	133	91.1	0.2212
**History of cardiac disease**	0	114	24.3	0	25	17.2	0	48	26.9	0	41	28.1	0.0564
**Male donor**	0	251	53.5	0	78	53.8	0	100	56.2	0	73	50.0	0.5385
**Donor type**	0			0			0			0			
Living		74	15.8		24	16.5		27	15.1		23	15.7	0.9441
SCD		207	44.1		81	55.8		72	40.4		54	36.9	0.0024
ECD		188	40.1		40	27.6		79	44.4		69	47.2	0.0009
**HLA-A-B-DR incompatibilities > 4**	0	124	26.4	0	40	27.6	0	39	21.9	0	45	30.8	0.1811
**Depleting induction**	0	253	53.9	0	96	66.2	0	91	51.1	0	66	45.2	0.0010
**BPAR in the first 2 months**	0	1	0.002	0	1	0.006	0	0	0	0	0	0	>0.9999
	NA	m	SD	NA	m	SD	NA	m	SD	NA	m	SD	p-value
**Recipient age (years)**	0	53.7	14.2	0	48.1	14.2	0	55.3	13.7	0	57.4	13.2	<0.0001
**Cold ischemia time (hours)**	0	754	444	0	711	444	0	780	446	0	766	439	0.3560
**Donor age (years)**	0	55.2	16.2	0	49.1	15.9	0	57.7	15.5	0	58.2	15.6	<0.0001
**Donor creatinine (ml/min/m²)**	0	90	55	0	98	71	0	89	51	0	84	40	0.0962

ECD, expanded criteria donor; HLA, human leucocyte antigens; NA: not available (missing); SCD, standard criteria donor; SD, standard deviation, C0/D : Tacrolimus trough level/Tacrolimus dose
